# Airborne pathogens diffusion: A comparison between tracer gas and pigmented aerosols for indoor environment analysis

**DOI:** 10.1016/j.heliyon.2024.e26076

**Published:** 2024-02-15

**Authors:** Marco Puglia, Filippo Ottani, Nicolo’ Morselli, Simone Pedrazzi, Giulio Allesina, Alberto Muscio, Andrea Cossarizza, Paolo Tartarini

**Affiliations:** a Università di Modena e Reggio Emilia, Dipartimento di Ingegneria “Enzo Ferrari”, Via Pietro Vivarelli, 10-41125, Modena, Italy; b Università di Modena e Reggio Emilia, Dipartimento di Scienze Mediche e Chirurgiche Materno Infantili e dell'Adulto, Via del Pozzo, 71, 41124, Modena, Italy

**Keywords:** Airborne transmission, Covid-19, Tracer gas, Aerosol, Droplet nuclei, Droplets

## Abstract

The evaluation of airborne pathogens diffusion is a crucial practice in preventing airborne diseases like COVID-19, especially in indoor environments. Through this transmission route, pathogens can be carried by droplets, droplet nuclei and aerosols and be conveyed over long distances. Therefore, understanding their diffusion is vital for prevention and curbing disease transmission. There are different techniques used for this purpose, and one of the most common is the utilization of tracer gas, however, it has limitations such as the difference in size between the gas molecules and the respiratory droplets, as well as its incapability to take into account evaporation. For this reason, a new method for evaluating the diffusion of respiratory droplets has been developed. This approach involves the use of an ultrasonic emitter to release and disperse pigmented aerosols, and a colorimeter for the following quantitative evaluation. A comparison with the tracer gas technique has been carried out, showing for the pigmented aerosols methodology a response that is dependent on different relative humidity conditions, while there is no clear difference in the dispersion of tracer gas at high or low humidity.

## Highlights

• A new methodology has been devised to assess the diffusion of respiratory droplets.
• This proposed approach employs an ultrasonic emitter to disperse pigmented aerosols, complemented by a colorimeter for precise quantification of the deposited fraction.
• Conducted within a controlled environment featuring controlled thermo-hygrometric conditions, an experimental campaign took place in a test chamber.
• A comparison with the tracer gas technique was undertaken.
• The pigmented aerosolized droplet methodology demonstrated an higher accuracy across various relative humidity conditions.

## 1 Introduction

In late 2019, a novel human coronavirus (Betacoronavirus subgenus) appeared in Wuhan (Hubei), China, fast spreading worldwide causing the SARS-CoV-2/COVID-19 (severe acute respiratory syndrome coronavirus 2/coronavirus disease 2019) global pandemic [[1], [2], [3], [4]]. The most similar known human coronavirus to SARS-CoV-2 is SARS-CoV (approximately 80% RNA genome sequence identity) [5]. Various experimental studies confirmed the transmission of COVID-19 through the air, similar to SARS and other infectious diseases such as chickenpox, smallpox, tuberculosis, measles, and influenza [1,6]. The airborne route refers to the transmission of diseases or pathogens caused by the dissemination of aerosols, droplets, droplet nuclei or other particles by an infected individual, by means of sneezing, coughing, or speaking. These particles, when suspended in air, can remain infectious for a long time and travel long distances, and therefore can be inhaled by a susceptible individual [5,7]. Concerning COVID-19 pandemic, aerosol transmission was proven to be the dominant contagion mechanism in multiple cases, such as the restaurant in Guangzhou (China) and the choir rehearsal in Valley Chorale (USA) [8]. Another study further proved that aerosol transmission could occur even without direct person-to-person contact, as it happened in Christchurch (New Zealand), during a hotel-managed isolation quarantine [9]. In fact, from an epidemiological perspective, preventing airborne transmission stands out as one of the most challenging problems in infection control [7], and hence, these outbreaks require a multidisciplinary approach that takes into account the various factors of the spread, such as the role of the indoor air environments [6]. To fulfill this task, a methodology for the tracing of airborne pathogens carried by human-generated droplets was developed and tested. A patent involving the proposed methodology has been granted (industrial patent number 102020000032021 [10]). The methodology aims at showing the distribution of potentially infectious droplets in a certain indoor environment in order to provide relevant information that can be used for multiple uses such as: implementation of proper ventilation strategies and physical distancing, validation of exposure strategies or ambient arrangement, aiding numerical simulations etc. This methodology utilizes pigmented aerosolized droplets (*PAD* from now on) emitted through an ultrasonic device to mimic and trace the respiratory droplets exhaled by a human. A preliminary version of this methodology was first presented in a previous work and was tested in a university classroom, successfully demonstrating that a significant portion of small droplets do not settle near the emission site [11]. Instead, they either remain suspended in air for an extended period or travel considerable distance. Furthermore, in case the ventilation systems provide a very low air flow velocity inside the room, as measured in the studied classroom, their contribution to altering the dispersion of the droplets is extremely modest [11].

### 1.1 The contribution of ventilation in infection transmission

Heating, ventilating, and air conditioning systems (HVAC) are responsible for providing healthy and comfortable indoor environments [6,12]. The purpose of ventilation is conveying and introducing outdoor or treated air into a building through either natural or mechanical techniques [6,12]. It is widely acknowledged as one of the most important engineering approaches employed to manage the spread of infectious particles indoors [7,13]. The investigation of droplets and aerosols behavior in association with indoor ventilation and possible infection transmission has been widely discussed [14]. Over the history of HVAC systems, a multitude of air distribution techniques have been developed (e.g. mixing ventilation, displacement ventilation, personalized ventilation etc.) [13]. Each of these approaches comes with its own set of advantages and disadvantages. They may be more suitable for a particular application than others, and in addition, they can have a different effect on airborne transmission risk. A possible strategy to reduce cross infection is the implementation of personalized ventilation, nevertheless, this solution can heighten the risk when the air inlet is in proximity to an infected person as a result of the greater dispersion of the exhaled contaminated particles [13]. Avoiding air recirculation is one of the most useful strategies that can be put into practice to reduce airborne transmission (especially during an epidemic or a pandemic). In cases where this is not possible, filtering the recirculated air and/or applying ultraviolet germicidal radiation can be valuable approaches to mitigate the risk [12]. In edge cases like hospital wards, the room pressure can be kept negative due to the possible presence of infectious individuals [6,12]. Also increasing the number of air changes per hour can be effective in reducing the diffusion of infectious diseases. However, even if the dilution effect on contaminated air is evident, the consequent higher air velocities in the room can increase the spread of infection. Moreover, while with high supply rate there is a reduction in the overall concentration of droplets in most areas of an environment, this decrease may be offset by the spread toward areas that were arguably safe in presence of low number of air changes per hour [12,13,15]. De Oliveira et al. [8] claim that to ensure a highly effective cleansing effect through ventilation, approximately 100 air changes per hour are necessary to obtain this result. This value is about one order of magnitude greater than hospital design settings, therefore it is almost impossible to be implemented using existing air management equipment and therefore other complementary measures are needed: face covering, occupancy reduction and increased distances between occupants [8,13].

### 1.2 Indoor environment analysis through aerosol tracing

Having a practical and reliable method for the evaluation of the transmission routes in indoor environments is crucial to limit the spread due to the strong lack of knowledge regarding the optimal ventilation design and conditions [16]. Literature reports different methods that can be used to mimic the motion of infectious aerosol droplets for the assessment of the airborne transmission diffusion. Lordly et al. [17] prototyped a novel artificial ‘cough’ generator that produces propylene glycol solution spray; in this case, a particle counter is necessary to collect the emitted aerosols. Zhang et al. [18] assembled a twin-aisle cabin mockup and used a tracer gas (namely sulfur hexafluoride) and di-ethyl-hexyl-sebacat (DEHS) particles. To measure the dispersion, they used a photo-acoustic multi-gas analyzer and an optical particle sizer [18]. However, both the cited substances do not experience evaporation (DEHS saturation pressure at 295 K is below 1 Pa [19]). The use of a tracer gas (often N_2_O and CO_2_) has the advantage of being relatively simple and that can be applied in various locations [13,20,21]. On the other hand, many different physical forces act on droplets with respect to gas molecules: gas molecules tend to bounce after impacting a solid surface while droplets are more prone to attach to the surface due to adhesive force [21]. Moreover, droplets are subject to the phenomenon of evaporation which progressively changes the characteristics of their motion once emitted. In the study of bio-aerosol dispersion (aerosol containing living material or that are originated from living organism), commonly used nebulizers include the collision nebulizer, Blaustein Atomizing Modules (BLAM), and the Sparging Liquis Aerosol Generator (SLAG) [22,23]. These systems provide aerosols with a mass median aerodynamic diameter below 3–4 μm [[24], [25], [26], [27]]. Only a Collison nebulizers can be operated to generate larger droplets, with a diameter up to tens of μm [28,29]. In the methodology presented here, an ultrasonic emitter is used to aerosolize and spread particles, which can be used to properly mimic the human-generated droplet dispersion behavior. This device has been chosen for the best performance in transporting the pigment (food-grade ink) used to trace the motion of dispersed droplets. Particles emitted by ultrasonic emitter have usually a diameter in the range of 1–10 μm [30,31] that is a size consistent with the ones typically exhaled during cough or speech (diameter ≤20 μm) and this particles can float in air for more than 60 min [1]. For these droplets, and especially for the ones smaller than 10 μm, gravity and inertia play a weak role on their dynamics, and they can travel for longer distances, while on the other hand large droplets follow a ballistic trajectory, falling within a limited space [5]. Furthermore, droplet size is not constant over time, but varies due to the evaporation phenomenon and the reduction speed increases as they decrease in size [32]. Droplet nuclei are the residues of evaporated droplets that reached the equilibrium with ambient air, generated by respiratory activities ranging from 1 to 5–10 μm and. Therefore, if a droplet does not sediment before drying it becomes a droplet nucleus [5,21,33]. Because of the small size of droplet nuclei, Stokes' number becomes much lower than 1, therefore the particles tend to follow the motion of the air [34,35]. The methodology presented in this work is focused on small droplets because of their ability to transport viable viruses to significant distances and therefore they are much more dangerous than large droplets which fall quickly to the ground as shown by the well-known Wells infectious transmission evaporation-falling curve [36]. In fact, SARS-CoV-2 virus can remain viable and infectious in aerosols for hours (with a half-life of more than an hour), and on surfaces up to days (e.g. half-life on 5.6 h on stainless steel and 6.8 h on plastic). Additionally, mucus and surfactant that remain after the evaporation process can protect and prolong the viability of the virus [2,5].

### 1.3 Droplet motion

**Fig. 1:**
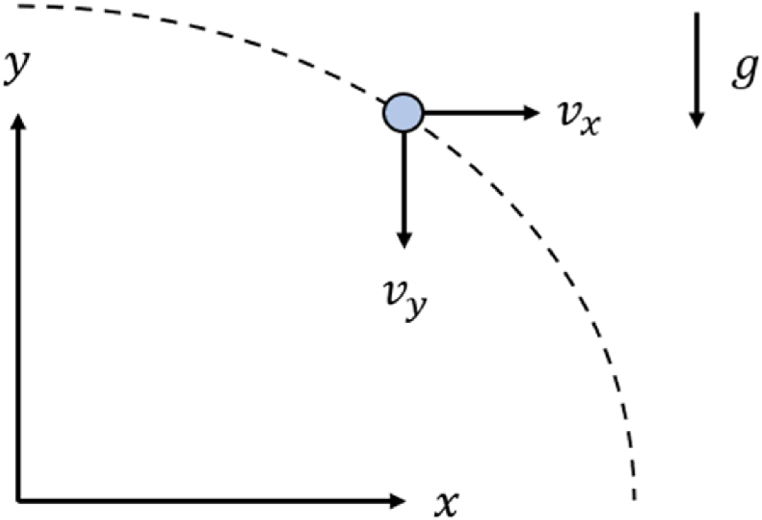
Droplet trajectory.

In this section, the basic equations of the droplet motion are presented to contextualize the research and underscore the significance of evaporation. In situations where the Stokes’ law is reliable (Reynolds number up to 0.1), the total force of the air acting on a respiratory droplet decomposed along the x and y directions (Fig. 1), can be written as:

(1) $$F_{y}=\frac{4}{3}\pi r^{3}\left(\rho_{a}-\rho_{d}\right)+6\pi \mu rv_{y}$$

(2) $$F_{x}=-6\pi \mu rv_{x}$$

Where *r* is the droplet radius, *ρ*_*a*_ and *ρ*_*d*_ are the densities of air and of the droplet, respectively, μ is the air dynamic viscosity. *V*_*x*_ and *v*_*y*_ are the droplet velocities along the x and y axes, with *v*_*x*_ > 0 and *v*_*y*_ < 0 [37].

From equation (1), it is evident that kinetic force *6πμrv*_*y*_ is linearly dependent to the radius, while weight force varies as the cube of the radius, for this reason if the radius halves, the kinetic force halves as well, but the weight changes with the cube root, therefore smaller droplet tend to float for longer time. Concerning the law of motion along the x axis, it can be written as:

(3) $$L_{x}=v_{x}t+\frac{1}{2}a_{x}t^{2}=v_{0x}t+\frac{1}{2}\frac{F_{x}}{m_{d}}t^{2}=v_{0x}t-\frac{1}{2}\frac{6\pi \mu rv_{x}}{m_{d}}t^{2}=v_{0x}t-\frac{1}{2}\frac{6\pi \mu rv_{x}}{\left(\frac{4}{3}\pi r^{3}\rho_{d}\right)}t^{2}=v_{0x}t-\frac{9\mu v_{x}}{{4r}^{2}\rho_{d}}t^{2}$$

From this equation it is evident that the smaller the radius, the more dominant the second term. Also, considering the forces acting in case where Stokes’ law it is not functional (equation (4) and (5)) it is possible to see a strong dependence of the motion of the droplet on its radius [38]:

(4) $$F_{y}=\frac{4}{3}\pi r^{3}\left(\rho_{a}-\rho_{d}\right)-\frac{1}{2}\rho_{a}Cv_{y}^{2}\pi r^{2}$$

(5) $$F_{x}=-\frac{1}{2}\rho_{a}Cv_{x}^{2}\pi r^{2}$$

Where *C* is the drag coefficient that depends on Reynolds number [39]. From what has been stated, it follows that droplet radius variation has a significant effect on droplet motion. Within this context, evaporation plays an important role on droplet trajectory. The decrease rate over time of a droplet radius *r* is described by the following formula [40,41]:

(6) $$\frac{dr}{dt}=\frac{-M_{L}D_{v,f}}{r\rho_{L}RT_{f}}\Delta p\left(1+0.276{Re}^{1/2}{Sc}^{1/3}\right)$$

Where *M*_*L*_ is the molecular weight of the considered liquid, *ρ*_*L*_ is its density, *D*_*v,f*_ is the diffusion coefficient for vapor molecules in the saturated film around the droplet, *T*_*f*_ is the average temperature in the film, Re and *Sc* are Reynolds' and Schimdt's numbers for the saturated film, *Δp* is the difference between the vapor pressure near the droplet and the vapor pressure in the ambient, and *R* is the universal gas constant. As can be seen from the equation, the lower the vapor pressure of the ambient (for the same saturated vapor pressure), the higher the radius decrease rate. Relative humidity is, therefore, a fundamental parameter in the dimension variation of the droplet, and consequently on its trajectory and settling time as seen from the previous equations [42]. In this paragraph, only the simple scenario of a single droplet was considered. However, even in a more complex situation, relative humidity plays a fundamental role, such as in the presence of a breath clouds and jet and puff phases, which characterize the periodicity of respiratory events [43]. Due to the reasons outlined, the use of a tracer gas to assess the dispersion of potentially infectious respiratory droplets may lead to misinterpretations of their motion. In this work, a comparison between the use of a tracer gas and the *PAD* alternative methodology was performed in a controlled indoor environment.

## 2 Materials and methods

**Fig. 2:**
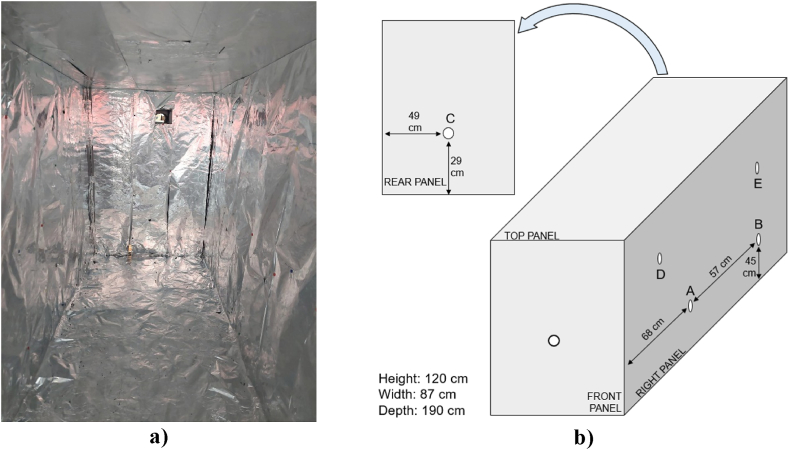
Test chamber built for the test, **a)** Picture of the inside **b)** Scheme.

The comparison was performed in a specifically built chamber used as an indoor environment where hygrometric conditions can be controlled (Fig. 2). In this experimental campaign, it was crucial to significantly elevate air humidity to facilitate a comparative analysis of the efficacy of two distinct tracing methods under varying humidity conditions, encompassing both low and high levels.

The side walls of the chamber are constructed with self-supporting material (extruded polystyrene blocks), 60 mm thick. Bottom and top layers are made of plastic material. The useable internal volume is 1.98 m^3^. The inner surfaces were covered with aluminum foils that were electrically grounded to avoid possible interaction between tracers and electrostatic charges. On the side walls, holes have been bored for the insertion of humidity and gas concentration probes. On the front panel, another hole can be used for injecting the *PAD* or the tracer gas, while the rear panel houses an extraction nozzle for extracting vapors and gases at the end of the tests, restoring the initial conditions. Eight tests were performed, in particular consisting of two repetitions at medium and two repetitions at high humidity with argon used as tracer gas, and two repetitions at medium and two repetitions at high humidity with *PAD*.

### 2.1 PAD tests

The aerosol emission phase is performed through a Levoit LV550HH ultrasonic humidifier. The operation frequency of its ultrasonic atomizer of this device is 1.7 MHz, hence it is possible to assume that the average diameter of the small droplets emitted is about 6 μm [30,31], a dimension in line with the droplet size investigated also in other works on airborne transmission. The water tank is filled with a mixture of deionized water (99% w/w) and non-toxic food-grade ink (1% w/w), following the proportion between liquid and solid matter in human saliva [44]. Using water instead of other substances with different saturation pressure allows to mimic the evaporation of human-generated droplets. The emitter is equipped with a fan to propel the air filled with droplets and a pipe to guide the flow. At the setpoint chosen, it emits about 300 mL every hour and the air flow velocity at the outlet of the pipe is 1 m/s, similar to the velocity of the air exhaled from a mouth during the breathing activity [45]. This flow velocity was measured using a vane probe (Ø 16 mm, digital), accuracy ±(0.2 m/s + 1 % of the measured velocity) connected to a *Testo 440 – Air velocity and IAQ* measuring instrument. The dispersion of the small droplets in the chamber or in another indoor environment is traced by evaluating the quantity of pigmented aerosols that deposits on the surfaces displaced in the environment. This can be performed by placing A4 white paper sheets in the zone of interest and measuring their difference in color before and after the emission. The A4 paper sheets used as detectors do not disturb the flow, unlike a particle counter, which draws in a certain amount of air during operation. A *Portable NH300 Colorimeter* [46] with an aperture diameter of 8 mm was used to measure the *L*, *a*, and *b* parameters of the *CIE 1976 L × a × b* color space. In this way it is possible to numerically evaluate the color difference [47]. This difference *ΔH* was defined as:

(7) $$\Delta H=\sqrt{{\Delta L}^{2}+\Delta a^{2}+\Delta b^{2}}=\sqrt{{\left(L_{1}-L_{2}\right)}^{2}+{\left(a_{1}-a_{2}\right)}^{2}+{\left(b_{1}-b_{2}\right)}^{2}}$$

**Fig. 3:**
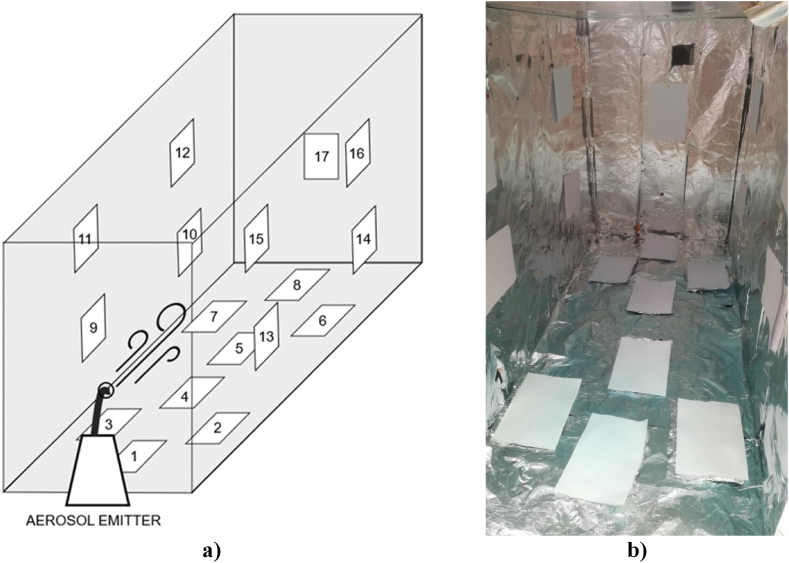
a) Sheets positioning **b)** Sheets after the *PAD* test.

The color was measured in 9 spots for each sheet. 17 sheets were positioned inside the chamber as depicted in Fig. 3 after a preliminary color evaluation.

The humidity inside the chamber was measured with a high-precision humidity/temperature probe, accuracy ±0.6%, connected to the *Testo 440* instrument. The probe was inserted into the chamber through the various holes on the left-side wall, on the right-side wall and on the rear. The ultrasonic emitter was placed outside the camber, with the pipe positioned to insufflate into the chamber the plume composed of *PAD*. The emitter was turned on for 3 min, followed by a 60-min settling period before opening the chamber. The air of the chamber was changed activating a fan and the sheets were collected and left to dry for 24 h. Afterward, their color was measured. The color variation (*ΔH*) was correlated to the mass of the droplets deposited (*ΔM*) through the following equation obtained in the calibration phase described in Appendix A:

(8) ΔM=0.033311590596353×ΔH+0.052399516132895

*ΔM* can be considered zero below a certain value of *ΔH*. The test was then repeated at a similar humidity level (around 47%). Subsequently, another humidifier was used inside the chamber to increase the relative humidity inside it. Two additional tests were repeated with a humidity level approximately 20% higher than the first two.

### 2.2 Tracer gas

**Fig. 4:**
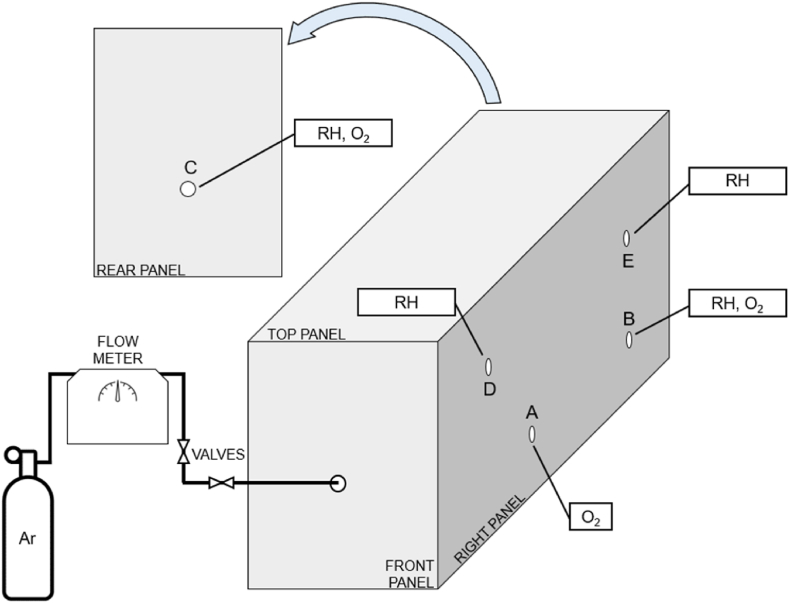
Tracer gas tests.

The dispersion of a tracer gas in the chamber was evaluated using argon (Ar, atomic number equal to 18, atomic weight equal to 39.948). Argon is a colorless and odorless very inert gas, commercially available at affordable price [48]. An argon cylinder was connected to the chamber through a pipe equipped with a *G4* volume flow meter and a series of valves to regulate the flow (Fig. 4).

To measure the diffusion of argon a T*esto 350 – Portable emission analyzer* equipped with a T*esto 350 – Control Unit*, *Flue gas probe* and a *O*_*2*_ *gas sensor* was used. The *O*_*2*_ probe was sequentially placed in three holes in the chamber panels, starting from position 'A', then moving to position 'B', and finally to position 'C' (at the rear). Humidity content was measured before the test using the humidity probe, following a similar procedure to the *PAD* test. By tuning the valves, an argon volume flow of approximately 2 L per minute was streamed into the chamber. The oxygen concentration was initially measured at position 'A'. It was then measured again after 5 min in the same position 'A', and then moving the humidity probe every 3 min in the positions: 'B', 'C', 'A', 'B', and finally 'C'. The first two tests were carried out without altering the ambient relative humidity, while the second two tests were performed increasing the humidity with a humidifier by approximately 20%, similarly to what was done in the previous *PAD* test. After each test, the fan placed in proximity to the ceiling of the chamber was activated for various minutes to restore the initial conditions, including both humidity and air composition. Two indicators were used to evaluate the tracer gas behavior in the various situations. The first one was the oxygen concentration in the three positions at various times of detection. The second one was the percentage variation of the oxygen concentration at different positions. This variation was weighted on the argon volume (*V*_*Ar*_) streamed into the chamber, namely *ΔO*_*2*_*/(O*_*2*_*∙V*_*Ar*_*)*. The scope of this second indicator is to properly take into account both the difference between the *O*_*2*_ concentration in cases with low humidity (higher *O*_*2*_ concentration) and high humidity (lower *O*_*2*_ concentration), as well as the difference due to a slightly different volume of argon flowed into the chamber.

## 3 Results

### 3.1 PAD results

**Fig. 5:**
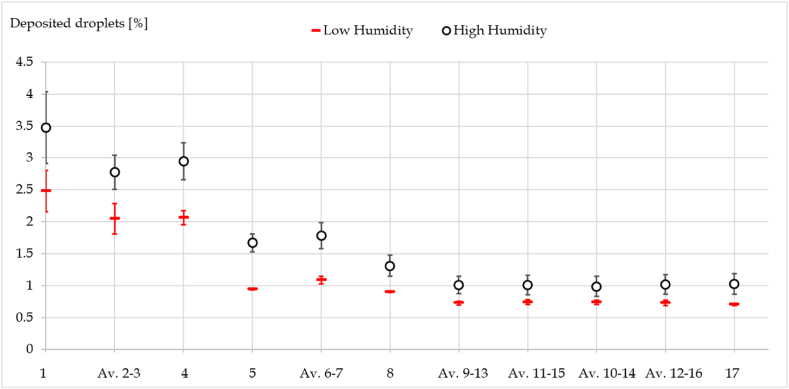
Mass fraction of droplets deposited on the various sheets in chamber.

The results obtained with the *PAD* methodology are presented in Fig. 5. The y-axis indicates the mass fraction deposited on each paper sheet (x-axis) compared to the amount of droplets emitted. For clarity, the graph displays the average value of two sheets (e.g. Av. 2–3) when there are 2 sheets at the same distance from the emitter.

It is evident that during high humidity cases, a higher amount of droplets deposited on the papers, while for low humidity the deposited fraction is lower. Furthermore, there is a perceptible difference between the two high humidity cases. In particular, High Humidity 1 (67.1%) has a higher deposition compared to High Humidity 2 (65.7%). These results are in line with literature, in fact: sedimentation time increases with mass reduction due to evaporation. Hence, a droplet that almost completely evaporates during its trajectory remains airborne as a droplet nucleus for a long period [49].

### 3.2 Tracer gas results

**Fig. 6:**
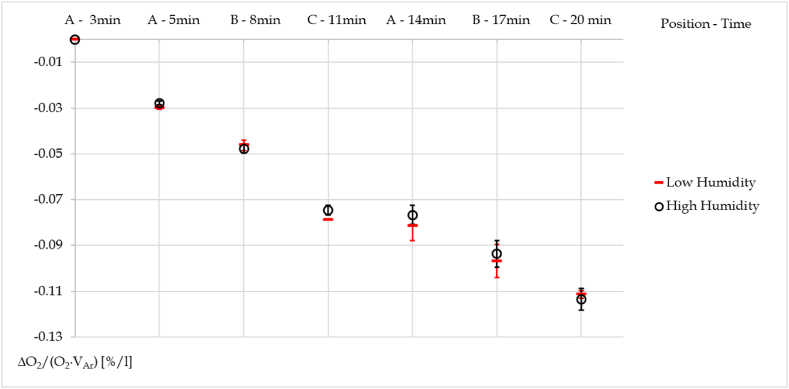
Weighted variation of oxygen concentration trend in the chamber.

The measurements performed with a tracer gas are summarized in Fig. 6. It depicts the percentage variation of the oxygen concentration in the chamber (weighted on the argon volume streamed) at various positions over time.

As can be seen from the graph, the detection does not reveal a straightforward link between dispersion and humidity. In fact, it is evident that there is no clear difference in the dispersion of argon flow between the low humidity cases and the high humidity ones.

## 4 Discussion

The experimental campaign carried out with the two tracing methods showed a remarkable difference in the dispersion of *PAD* between high humidity and low humidity conditions, whereas the tracer gas exhibited similar behavior under different humidity levels. Considering that relative humidity plays a crucial role in saliva droplet transportation [50], an effective way to measure the diffusion of human respiratory droplets should be sensible to ambient hygrometric conditions. Furthermore, *PAD* methodology properly predicted the higher deposition at high humidity, due to the lower evaporation rate. In this case the focus was on the tracing mechanism rather than the simulation of a specific breathing activity. Nevertheless, there are no specific constraints preventing the application of a similar approach with *PAD* to simulate coughing or speaking by varying the airflow velocity. Furthermore, a variation of the oscillation frequency of the ultrasonic emitter would enable the alteration of the median diameter of the emitted droplet, and hence it is possible to expand the range of the study. When applied in an indoor environment to assess potential transmission routes, this methodology should be conducted without introducing biological hazards. Therefore, in this study, the capability of carrying viable pathogens was not considered. This is because the primary objective is to trace the carriers of the pathogens, namely human-generated droplets, without evaluating the vitality of pathogens in different conditions. Larger pathogens, such as bacteria and fungi, are usually found in larger particles, while smaller particles can serve as carriers for viruses [51]. Considering that the dimensions of SARS-CoV-1 and 2 have an average envelope diameter of 80–90 nm and 60–140 nm, respectively [52,53], and the typical size of the H1N1 influenza virus ranges from 80 to 120 nm [54], it can be inferred that this methodology is more suitable for studying pathogens carried by small droplets, such as viruses, rather than larger bacteria or fungi. In the development of *PAD* methodology, the primary focus was on smaller droplets because they are the ones that can travel longer distances and remain suspended for an extended period [5,7]. Although ultrasonic emitters can potentially produce droplets up to tens of micrometers [31], this range has not been tested yet. An additional potential limitation of this methodology arises from the requirement for a new calibration phase when the substrate used for collecting pigmented aerosols is altered. For instance, using A4 papers from different brands may introduce variations in their color, potentially influencing the results if a recalibration is not performed.

## 5 Conclusions

This study presents a novel and easily reproducible methodology for investigating the diffusion of respiratory droplets in indoor environments. By employing an ultrasonic emitter to disperse pigmented aerosolized droplets, followed by their detection using a colorimeter, it was possible to discern distinct behaviors of particles under different relative humidity conditions. This capability has not been detected using the conventional tracer gas method, as expected. This is because gases do not undergo evaporation, a critical phenomenon that must be considered for a precise analysis of droplet dispersion. Implementing measures such as replicating thermal plumes and regulating the flow rate to mimic human exhalation rate, it becomes feasible to accurately simulate droplet emissions from a human. This allows an assessment of indoor air quality by evaluating the effectiveness of ventilation systems with consequent risk identification, and the implementation of necessary corrective actions.

## Funding statement

This work was supported by “10.13039/100007383Fondo di Ateneo per la Ricerca 2021” for the financial of departmental development plans in the field of research. Protocol No. 2424, dated July 08, 2021, Department of Engineering “Enzo Ferrari”, Università degli Studi di Modena e Reggio Emilia.

## Data availability

Data will be made available on request.

## CRediT authorship contribution statement

**Marco Puglia:** Writing – review & editing, Writing – original draft, Visualization, Validation, Supervision, Resources, Methodology, Investigation, Funding acquisition, Formal analysis, Data curation, Conceptualization. **Filippo Ottani:** Writing – review & editing, Writing – original draft, Visualization, Methodology, Investigation. **Nicolo’ Morselli:** Writing – review & editing, Investigation, Conceptualization, Methodology, Writing – original draft. **Simone Pedrazzi:** Writing – review & editing, Supervision, Resources, Investigation, Funding acquisition, Conceptualization. **Giulio Allesina:** Writing – review & editing, Supervision, Conceptualization, Writing – original draft. **Alberto Muscio:** Supervision, Resources, Conceptualization. **Andrea Cossarizza:** Supervision, Resources, Investigation, Conceptualization. **Paolo Tartarini:** Writing – review & editing, Supervision, Resources, Conceptualization.

## Declaration of generative AI and AI-assisted technologies in the writing process

During the preparation of this work the authors used ChatGPT-3.5 in order to improve language and readability. After using this tool/service, the authors reviewed and edited the content as needed and take full responsibility for the content of the publication.

## Declaration of competing interest

The authors declare the following financial interests/personal relationships which may be considered as potential competing interests: Marco Puglia, Nicolo’ Morselli, Giulio Allesina, Simone Pedrazzi, Alberto Muscio, Andrea Cossarizza, Paolo Tartarini are also inventors of the patent related to the proposed PAD methodology (industrial patent number 102020000032021 [10]).

## Figures and Tables

**Fig. A1:**
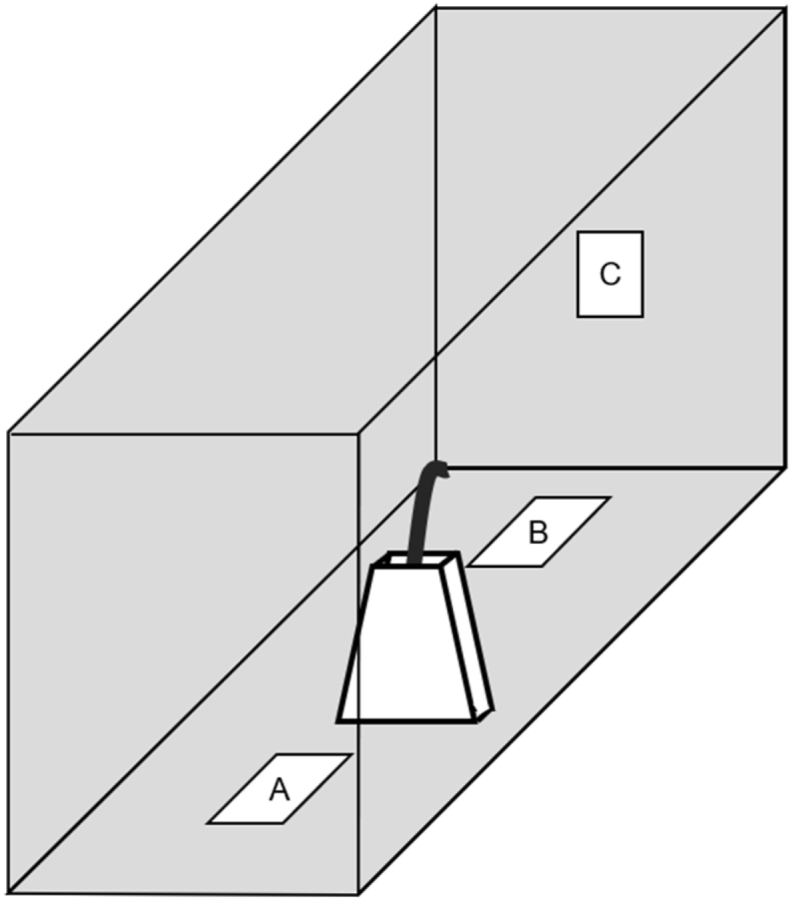
Calibration phase scheme

**Fig. B1:**
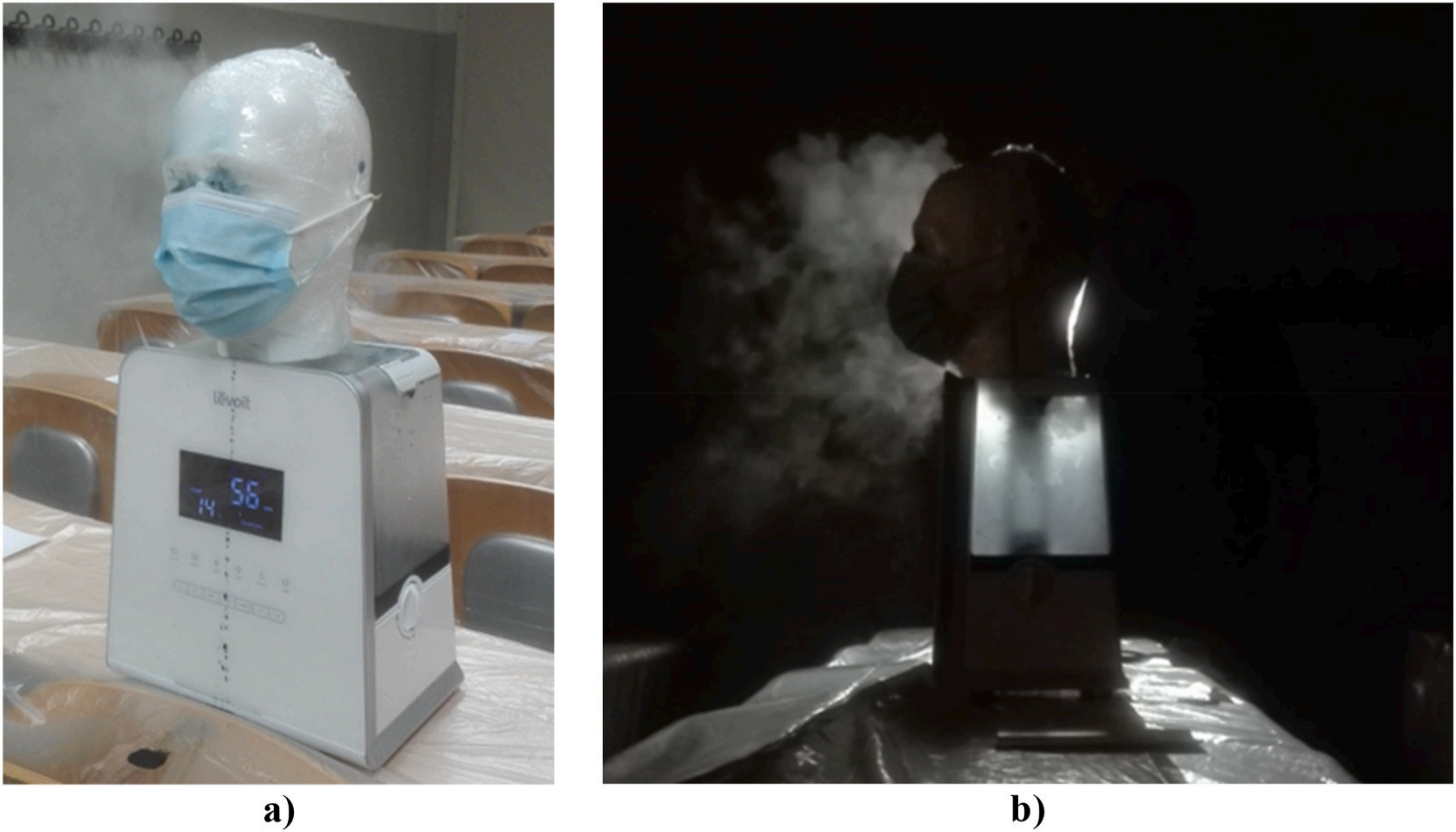
Tracing test with surgical mask

**Fig. B2:**
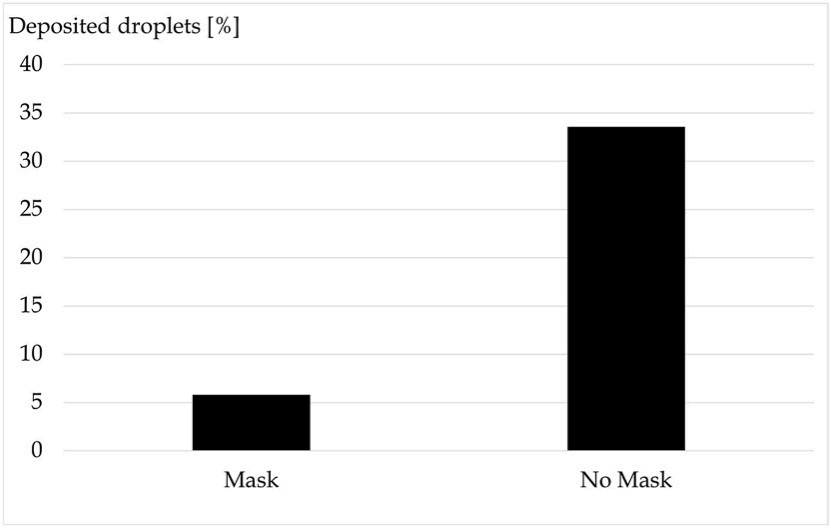
Comparison of the deposited fractions with and without surgical mask

## Appendix A Calibration phase

The calibration phase was carried out in a chamber with smaller dimensions compared to the one used for the test (0.6 m × 0.6 m × 1 m). Even in this case aluminum foils were used to cover up the wall and electrically ground the chamber to avoid electrostatic forces. This calibration aims to correlate the weight of the droplets deposited on the paper sheet and its color variation. The procedure was the following: three paper sheets were evaluated measuring their weight and their color (namely the L × a × b parameter with the *Portable NH300 Colorimeter*) then they were placed inside the chamber with the aerosol emitter as depicted in Fig. A1.

The chamber was sealed, and the emitter was activated for 30 s. Subsequently, the sheets were promptly removed from the chamber and weighed. It was hypothesized that due to the elevated moisture levels within the confined space, any droplets deposited on the sheets had insufficient time to evaporate, considering the weight loss negligible. The paper sheets were then left to naturally dry for 24 h, after which their color was assessed using the colorimeter. This process was repeated for durations of 60 and 90 s, in particular four times for 30 s, four times for 60 s, and twice for 90 s. A linear interpolation was then applied to correlate the weight increase (and hence the deposited droplets) with the color variation.

## Appendix B Validation test

The tracing tests were repeated positioning a surgical mask in front of the emitting pipe, fixing it on the polystyrene head, to reproduce the placement and the adherence of the mask on a human face (Fig. B1).

The goal was to test the effectiveness of the mask with the adopted methodology, in order to compare the obtained results with those found in literature to verify if the methodology can predict the mask protection factor. The comparison between the deposited droplets, measured with and without a surgical mask, is reported in Fig. B2.

As can be seen in Fig. B2, the amount of deposited aerosols and droplet nuclei measured in the test with the surgical mask is considerably lower, in particular a reduction of 78.2 % was reported. This reduction aligns with the 75 % decrease in surgical mask outward emission rate decrease reported in literature [55]. The ability to detect this variation is an advantage of this method compared to gas tracers. This denotes a further similarity in the behavior of aerosols and droplet nuclei exhaled by a breathing person and emitted by the ultrasonic emitter.
